# Hantaviruses: seroprevalence and risk factors among humans in Achaia prefecture, Greece

**DOI:** 10.1186/1471-2334-14-S2-P91

**Published:** 2014-05-23

**Authors:** George Panos, Maria Sargianou, Anna Papa, Charalambos Gogos

**Affiliations:** 1 Division of Infectious Diseases, Department of Internal Medicine, Patras University General Hospital, Patras, Greece; 2 Department of Microbiology, Medical School, Aristotle University of Thessaloniki, Thessaloniki, Greece

## Introduction

Inhalation of hantavirus-contaminated aerosol of rodents’ excreta can cause in humans hemorrhagic fever with renal syndrome (HFRS) in Eurasia and Hantavirus pulmonary syndrome in the Americas. In Greece, 1-5 cases are being diagnosed annually, mainly in the northern and western part. After the recent diagnosis of a hantavirus infection in Achaia prefecture (Peloponesse), a seroepidemiological study was designed to determine seroprevalence in the local population and to assess factors playing a role in the acquisition of hantavirus infection.

## Materials and methods

Between March-July 2012, 207 serum samples were prospectively collected. The participants were randomly selected among apparently healthy individuals, referred for routine blood testing or for blood donation to Patras University General Hospital and to healthcare centers of the 5 munipalities of Achaia. Sampling was performed and a questionnaire regarding demographics and potential risk factors was completed after a written consent had been obtained.

Serum samples were tested for hantavirus IgG antibodies by ELISA (Anti-Hanta Virus Pool 1 “Eurasia” ELISA IgG, Euroimmun, Lübeck, Germany), according to the manufacturer’s instructions. The data were statistically analyzed using SPSS 20.0 (IBM, Chicago, Illinois, USA).

## Results

In total, 20/207 (9.66%) serum samples tested IgG positive for hantavirus, with seropositivity rates ranging from 0-18.5% among municipalities; a local seroprevalence maximum was encountered in one of the municipalities (5/27, 18.5%). Univariate analysis showed that age (OR 1.036, 95%CI 1.011-1.062), peridomestic sighting of rodents (<200m radius from residence, OR 4.538, 95% CI 1.019-20.219) and ownership of a storing shed (OR 2.892, 95%CI 1.004-8.327) were statistically significant risk factors.

## Conclusions

An unexpectedly high hantavirus seroprevalence was observed in Achaia, indicating the presence of CCHFV in the region and the possible exposure of the habitants to hantaviruses. Studies in rodents are needed to identify the circulating strain(s), which could lead to a better understanding of the epidemiology of hantaviruses in southwestern Greece. Clinicians should include hantavirus infections in the differential diagnosis of acute febrile cases accompanied by renal impairment and/or hemorrhagic manifestations.

